# Supplementary material for: TACC3–ch-TOG track the growing tips of microtubules independently of clathrin and Aurora-A phosphorylation
Source: Biol Open. 2015 Jan 16;4(2):170–9. doi: 10.1242/bio.201410843 (PMC4365485; doi:10.1242/bio.201410843)
Supplement: Supplementary Material [file supp_bio.201410843_bio.201410843-s1.pdf]

Biology Open (2015) 000, 1–10 doi:10.1242/bio.201410843

## Supplementary Material

**Cristina Gutiérrez-Caballero et al. doi: 10.1242/bio.201410843**

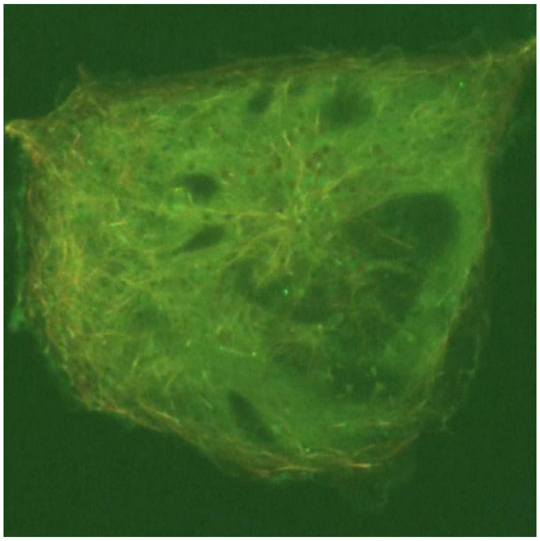

**Movie 1. A video of GFP-TACC3 expressed in an interphase RPE1 cell stably expressing EB1.** Live-cell imaging was done using a spinning disk microscope with a 100× ~1.4 NA oil immersion objective lens. Images were captured every second for 1 minute (video playback is 10 fps). Cells were excited at 488 nm and 561 nm and images captured simultaneously with two cameras (Hamamatsu C10600-10B ORCA-R2). The same cell is shown in Fig. 1A.

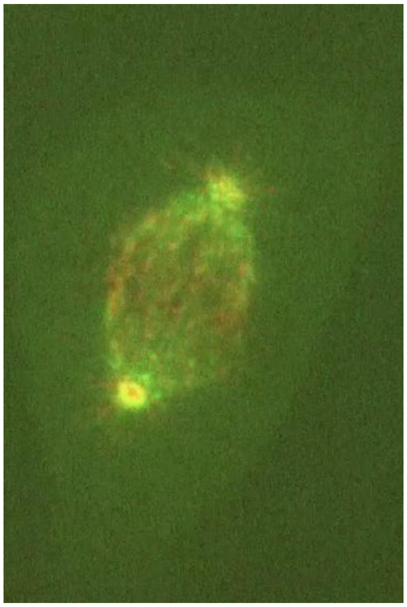

**Movie 2. A video of GFP-TACC3 expressed in a mitotic RPE1 cell stably expressing EB1.** Live-cell imaging was done using a spinning disk microscope with a 100× ~1.4 NA oil immersion objective lens. Images were captured every second for 1 minute (video playback is 10 fps). Cells were excited at 488 nm and 561 nm and images captured simultaneously with two cameras (Hamamatsu C10600-10B ORCA-R2). The same cell is shown in Fig. 1A.

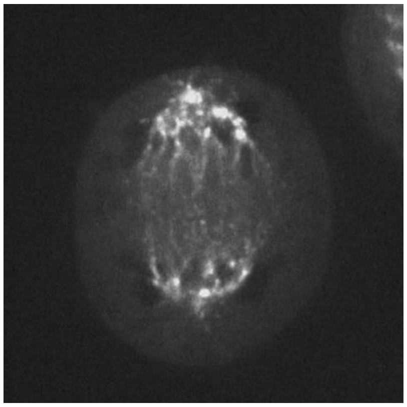

**Movie 3. A video of a HeLa Kyoto cell in anaphase expressing TACC3-GFP under its endogenous promoter.** Live-cell imaging was done using a spinning disk microscope with a 100× ~1.4 NA oil immersion objective lens. Images were captured every second for 1 minute (video playback is 10 fps). The same cell is shown in Fig. 1C.

Biology Open
